# Glucagon-like peptide-1 reduces pancreatic β-cell mass through hypothalamic neural pathways in high-fat diet-induced obese rats

**DOI:** 10.1038/s41598-017-05371-4

**Published:** 2017-07-17

**Authors:** Hisae Ando, Koro Gotoh, Kansuke Fujiwara, Manabu Anai, Seiichi Chiba, Takayuki Masaki, Tetsuya Kakuma, Hirotaka Shibata

**Affiliations:** Department of Endocrinology, Metabolism, Rheumatology and Nephrology, Faculty of Medicine, Oita University, Yufu city, Oita 879-5593 Japan

## Abstract

We examined whether glucagon-like peptide-1 (GLP-1) affects β-cell mass and proliferation through neural pathways, from hepatic afferent nerves to pancreatic efferent nerves via the central nervous system, in high-fat diet (HFD)-induced obese rats. The effects of chronic administration of GLP-1 (7–36) and liraglutide, a GLP-1 receptor agonist, on pancreatic morphological alterations, c-fos expression and brain-derived neurotrophic factor (BDNF) content in the hypothalamus, and glucose metabolism were investigated in HFD-induced obese rats that underwent hepatic afferent vagotomy (VgX) and/or pancreatic efferent sympathectomy (SpX). Chronic GLP-1 (7–36) administration to HFD-induced obese rats elevated c-fos expression and BDNF content in the hypothalamus, followed by a reduction in pancreatic β-cell hyperplasia and insulin content, thus resulting in improved glucose tolerance. These responses were abolished by VgX and SpX. Moreover, administration of liraglutide similarly activated the hypothalamic neural pathways, thus resulting in a more profound amelioration of glucose tolerance than native GLP-1 (7–36). These data suggest that GLP-1 normalizes the obesity-induced compensatory increase in β-cell mass and glucose intolerance through a neuronal relay system consisting of hepatic afferent nerves, the hypothalamus, and pancreatic efferent nerves.

## Introduction

β-cell mass, which is determined by the product of the number and size of pancreatic β-cells, is tightly controlled to maintain glucose levels within a normal range. β-cell mass increases along with an increase in demand for insulin induced by a pathological change such as obesity or pregnancy^1, 2^. Human obesity can result in an ~50% increase in β-cell mass, and these obesity-induced compensatory changes in β-cell mass are controlled by increases in cell size and adjustments to the rate of β-cell proliferation to increase insulin levels and maintain normoglycaemia^3, 4^. Continued β-cell compensation for insulin resistance results in deterioration of β-cell function and is associated with decreased β-cell mass (due to apoptosis) in obese individuals, alterations that are essential features of the pathogenesis of type 2 diabetes mellitus^5^.

Peripheral and central glucagon-like peptide-1 (GLP-1) systems play an essential role in glycemic control and energy balance regulation. The GLP-1 receptor (GLP-1R) is expressed in the pancreas as well as in extra-pancreatic sites, such as the brain and peripheral nerve terminals in contact with the portal vein^6^. GLP-1 acts predominantly on β-cells to control glucose homeostasis and regulates pancreatic endocrine function, stimulating insulin secretion and inhibiting glucagon secretion^7^. Although the hepatoportal region is important for glucose sensing, it may also be vital for the integration of central and peripheral GLP-1 actions. Indeed, we previously reported that the hepatic afferent nerve mediates feeding and glucose homeostasis^8^.

Brain-derived neurotrophic factor (BDNF) is a member of the neurotrophic factor family, members of which play a key role in the regulation of neuronal survival, growth, and maintenance^9^. BDNF regulates feeding in obese rodent models of diabetes and modulates glucose metabolism via an anorectic effect as well as by modulating glucagon secretion from pancreatic α-cells^10–12^. We previously demonstrated that tropomyosin receptor kinase B (TrkB; the BDNF receptor) is localized to α-cells but not β-cells, and BDNF affects glucose metabolism by modulating glucagon secretion via the central and peripheral nervous system without affecting insulin secretion^13^.

Thus, in the present study, we investigated whether 1) acute intraperitoneal administration of GLP-1 (7–36) activates hypothalamic neurons through hepatic afferent nerves in non-obese rats, 2) chronic GLP-1 (7–36) treatment affects β-cell mass and proliferation and glucose metabolism via hepatic afferent/pancreatic efferent nerves in high-fat diet (HFD)-induced obese rats with insulin resistance and increased β-cell proliferation, and 3) chronic administration of liraglutide, a GLP-1R agonist, also affects β-cell mass and proliferation and glucose metabolism in a similar manner.

## Results

### Effect of GLP-1 (7–36) treatment on c-fos expression and BDNF content in the hypothalamus

**Figure 1:**
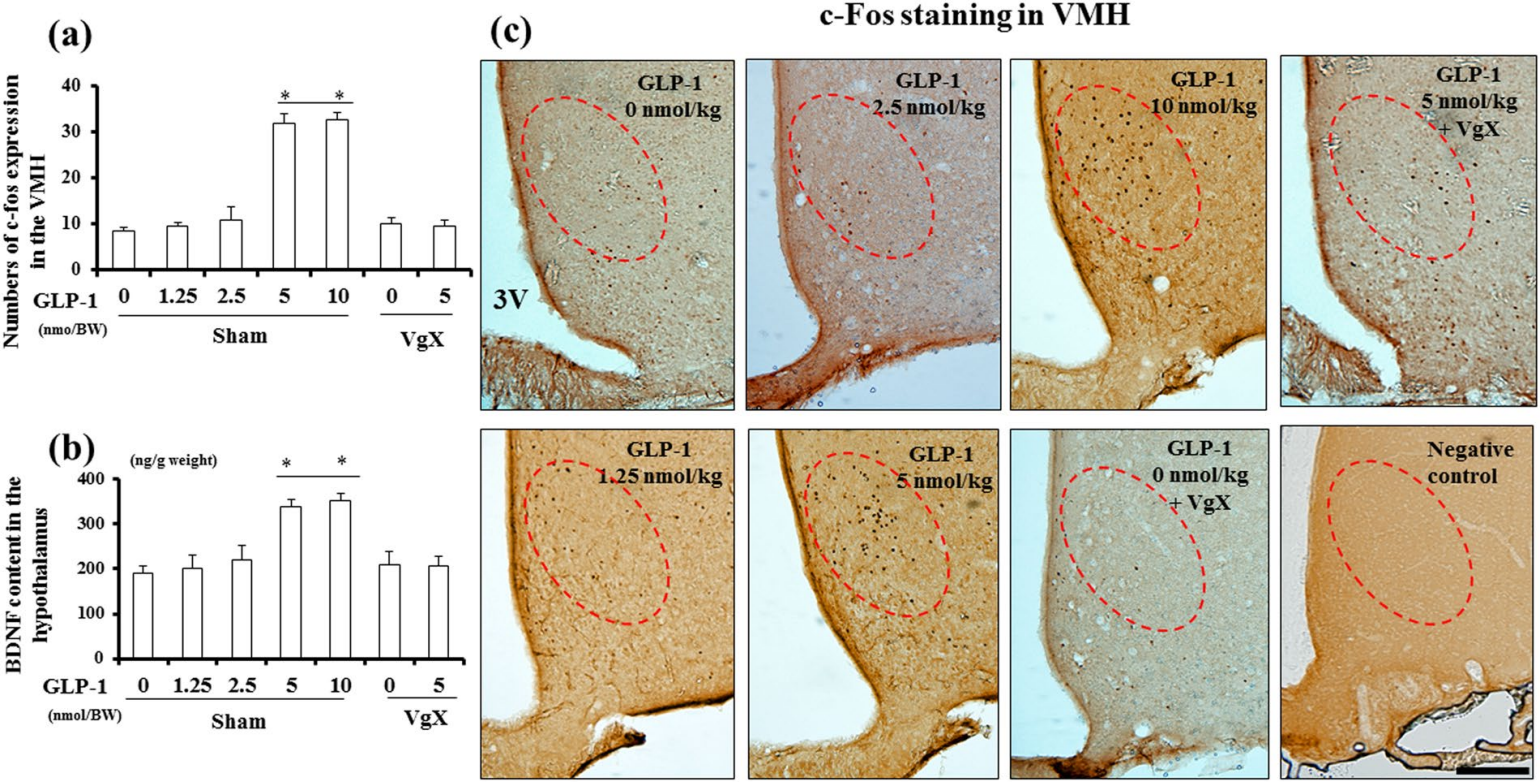
Acute administration of GLP-1 (7–36) at more than 5 nmol/kg BW increases c-fos expression and BDNF content in the hypothalamus through hepatic afferent nerves. (**a** and **b**) Quantification of c-fos expression in the VMH (**a**) and BDNF content (**b**) in the hypothalamus of each group (n = 6). (**c**) Representative c-fos staining in the VMH of brain sections from each group. Red circle indicates the VMH area in the hypothalamus. *p < 0.05 vs. GLP-1 (7–36) (0 nmol/kg), Treatments: GLP-1; intraperitoneal administration of GLP-1 (7–36), Sham; sham operation, VgX; hepatic afferent vagotomy. 3 V; third ventricle. Scale bar = 100 μm.

First, we examined the pattern of labelled cells in the dorsal motor nucleus (DMN) after transport of Fluorogold. Hepatic afferent vagotomy (VgX) decreased the number of Fluorogold-labelled cells in the DMN. Furthermore, only a few labelled cells in the DMN were observed in animals with a lesion of the dorsal sub-diaphragmatic trunk, indicating that the VgX was selectively performed (Supplementary Figure S3b). Figure 1 shows the number of c-fos-positive nuclei (a marker of activated neurons) in the brain sections. Administration of GLP-1 (7–36) at 5 and 10 nmol/kg but not at 0, 1.25, or 2.5 nmol/kg significantly increased c-fos expression in the ventromedial hypothalamus (VMH). VgX inhibited the elevation of c-fos expression in the VMH following administration of 5 nmol/kg GLP-1 (7–36), although VgX itself did not alter c-fos expression (Fig. 1a and c; n = 6 per group, *p < *0.05). These findings were also observed in the area of the paraventricular nucleus (PVN) (data not shown) but not in the arcuate nucleus (ARC) (Supplementary Figure S5a and S5c) or lateral hypothalamus (LH) (Supplementary Figure S5b). In addition, the acute administration of 5 and 10 nmol/kg GLP-1 but not 0, 1.25, or 2.5 nmol/kg GLP-1 (7–36) also increased BDNF content in the hypothalamus; the GLP-1-induced elevation of BDNF content was abolished by VgX, whereas VgX itself had no effect on BDNF content in the hypothalamus (Fig. 1b; n = 6 per group, *p < *0.05). Furthermore, we observed that the number of c-fos-positive cells was highly correlated with the hypothalamic BDNF content (Supplementary Figure S6, n = 42, r = 0.6148, *p < *0.05).

### Effect of VgX and SpX on body weight (BW), daily food intake, and fasting blood glucose during chronic GLP-1 (7–36) treatment

In Experiment Design 2, the BW of all HFD-fed groups was significantly higher than that of the standard diet (Standard)-fed group, and VgX did not alter the change in BW (Supplementary Figure 4a). In Experiment Design 3, the BW of all HFD-fed groups was also significantly higher than that of the Standard-fed group, except for at 1 week after sympathectomy (SpX) treatment (Supplementary Figure S4b). No significant differences were observed in daily food intake or fasting blood glucose among all HFD-fed groups. GLP-1 (7–36) did not affect the daily food intake or fasting glucose levels in both Experiment Design 2 and 3 (Supplementary Tables 1 and 2).

### Effect of VgX on c-fos expression and BDNF content in the hypothalamus during chronic GLP-1 (7–36) treatment

**Figure 2:**
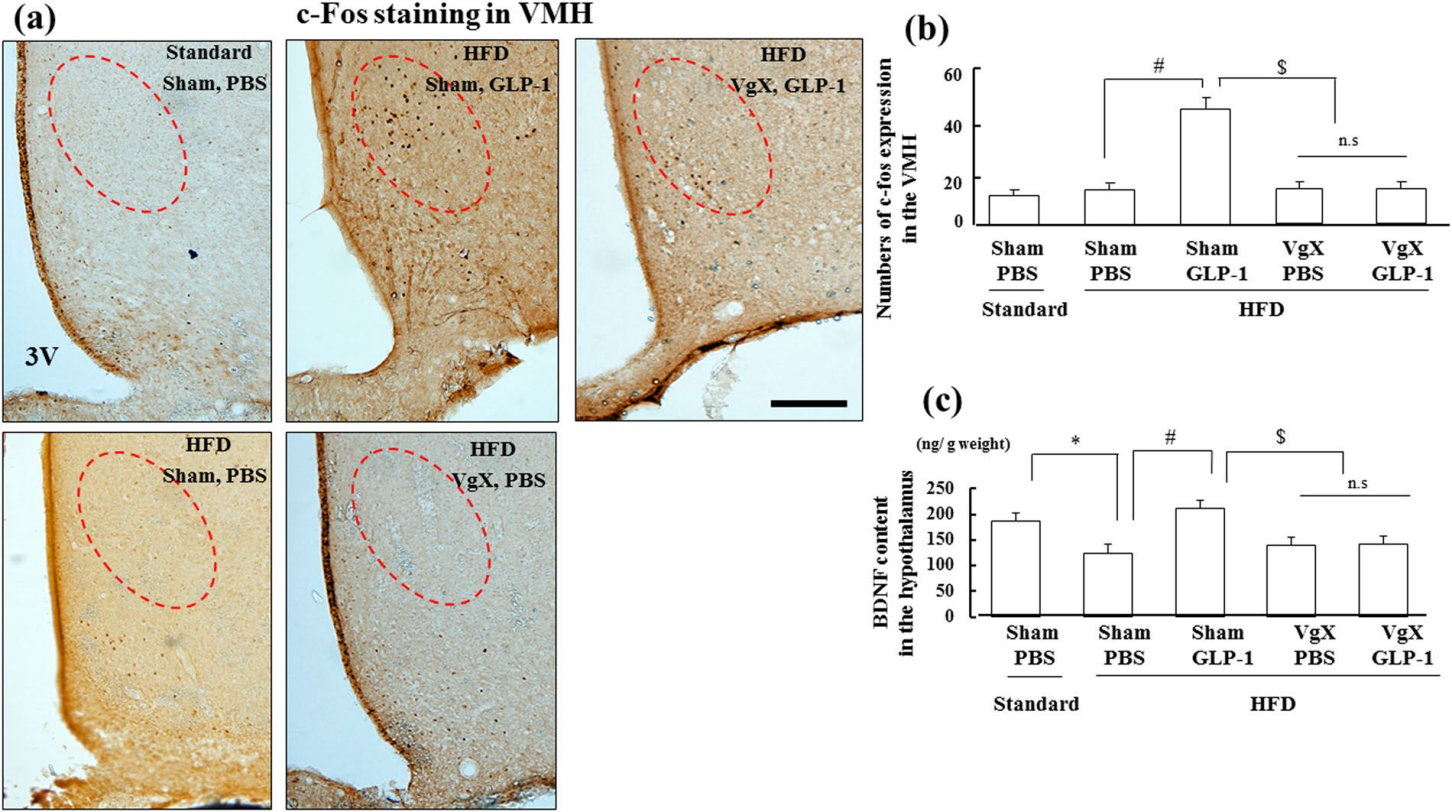
Chronic administration of GLP-1 (7–36) (5 nmol/kg BW) also increases c-fos expression and BDNF content in the hypothalamus through hepatic afferent nerves. (**a**) Representative c-fos staining of brain sections from each group. Red circle indicates the VMH area in the hypothalamus. (**b** and **c**) Quantification of c-fos expression in the VMH (**b**) and BDNF content in the hypothalamus (**c**) of each group (n = 6). *p < 0.05 vs. Standard (Sham, PBS) group, ^#^ *p* < 0.05 vs. HFD (Sham, PBS) group, ^$^ *p* < 0.05 vs. HFD (Sham, GLP-1) group. Treatments: PBS; intraperitoneal administration of PBS, GLP-1; intraperitoneal administration of GLP-1 (7–36), Standard; fed with standard diet, HFD; fed with high-fat diet, Sham; sham operation, VgX; hepatic afferent vagotomy. 3 V; third ventricle. Scale bar = 100 μm.

Chronic GLP-1 (7–36) (5 nmol/kg BW) treatment resulted in a significant increase in c-fos expression in the VMH compared to that in the HFD-fed PBS group, although HFD feeding alone did not influence c-fos expression. This GLP-1-induced alteration was suppressed by VgX (Fig. 2a and b; n = 6 per group, *p < *0.05). We observed no significant difference in c-fos expression in other hypothalamic nuclei, including the PVN area, among all groups (data not shown). In addition, BDNF content in the hypothalamus of the HFD-fed groups was significantly lower than that in the Standard-fed group. This obesity-induced decrease in BDNF content was restored by chronic administration of GLP-1 (7–36). Furthermore, this GLP-1-induced elevation was also inhibited by VgX, although VgX alone did not influence BDNF content (Fig. 2c; n = 6 per group, *p < *0.05).

### Effect of VgX on β-cell mass and proliferation in the pancreas during chronic GLP-1 (7–36) treatment

**Figure 3:**
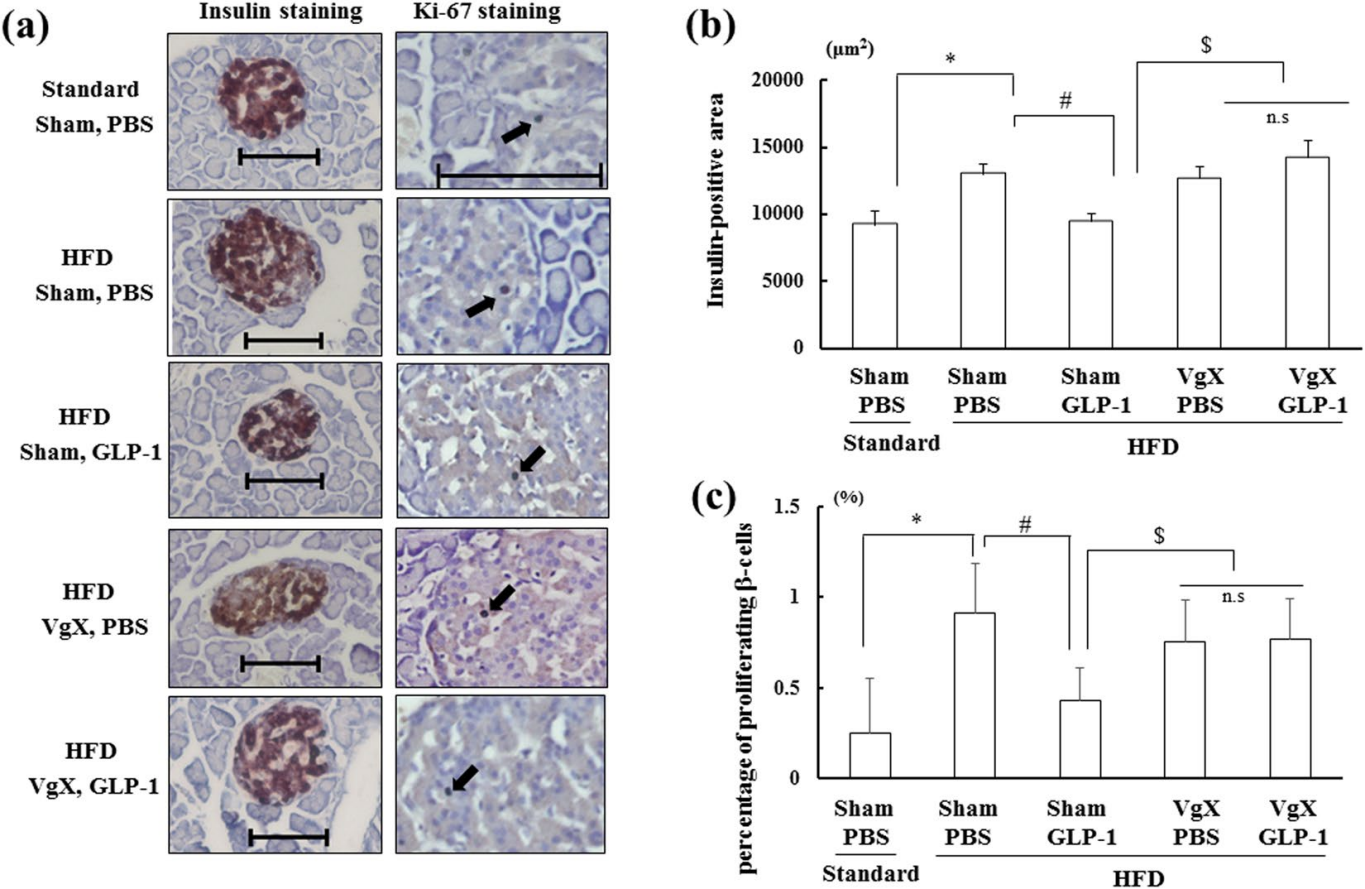
Chronic administration of GLP-1 (7–36) (5 nmol/kg BW) restores obesity-induced alterations in β-cell mass and proliferation in islets through hepatic afferent nerves. (**a**) Representative insulin staining (left row) and Ki-67 staining (right row) performed on pancreas sections from each group. (**b** and **c**) Insulin-positive area (**b**) and percentage of proliferating β-cells (**c**) in each group (n = 6). *p < 0.05 vs. Standard (Sham, PBS) group, ^#^ *p* < 0.05 vs. HFD (Sham, PBS) group, ^$^ *p* < 0.05 vs. HFD (Sham, GLP-1) group. Treatments: Standard; fed with standard diet, HFD; fed with high-fat diet, Sham; sham operation, VgX; hepatic afferent vagotomy, PBS; intraperitoneal administration of PBS, GLP-1; intraperitoneal administration of GLP-1 (7–36). Scale bar = 100 μm.

We investigated whether GLP-1 (7–36) administration affects pancreatic β-cell morphology and function. As expected, β-cell mass (the insulin-positive area; Fig. 3a and b) and the percent Ki-67-positive area, which corresponds to proliferating β-cells (Fig. 3a and c), were greater in the HFD-fed groups than in the Standard-fed group (n = 6 per group, *p < *0.05). The increase in these parameters in the HFD-fed groups was suppressed by chronic administration of GLP-1 (7–36) (Fig. 3, n = 6 per group, *p < *0.05). However, VgX preceding GLP-1 (7–36) treatment abolished these alterations, although VgX alone did not affect β-cell mass or the percentage of proliferating β-cells (Fig. 3). These data suggest that the suppressive effect of GLP-1 on obesity-induced alterations in β-cell mass is mediated by the hepatic afferent vagal nerves.

### Effect of SpX on β-cell mass and the intraperitoneal glucose tolerance test (IPGTT) during chronic GLP-1 (7–36) treatment

**Figure 4:**
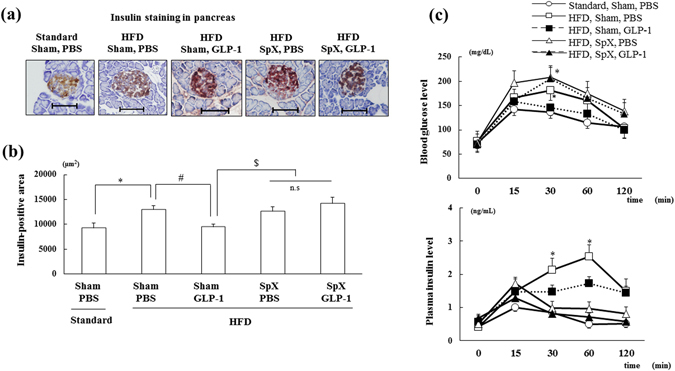
Chronic administration of GLP-1 (7–36) (5 nmol/kg BW) restores the obesity-induced enlargement of β-cell mass and improves glucose metabolism through pancreatic efferent nerves. (**a** and **b**) Representative insulin staining in islets (**a**) and insulin-positive area (**b**) in each group (n = 6). (**c**) Blood glucose (upper) and plasma insulin (lower) levels during the glucose tolerance tests in each group (n = 6). *p < 0.05 vs. Standard (Sham, PBS) group, ^#^ *p* < 0.05 vs. HFD (Sham, PBS) group, ^$^ *p* < 0.05 vs. HFD (Sham, GLP-1) group. Treatments: PBS; intraperitoneal administration of PBS, GLP-1; intraperitoneal administration of GLP-1 (7–36), Standard; fed with standard diet, HFD; fed with high-fat diet, Sham; preservation of pancreatic efferent sympathetic nerve with PBS administration, SpX; pancreatic efferent sympathectomy with 6-OHDA administration. Scale bar = 100 μm.

Furthermore, we investigated the role of pancreatic efferent nerves in vesicular monoamine transporter 2 (VMAT2)-positive nerve terminals, which are localized to the nerve endings of sympathetic neurons. 6-Hydroxydopamine (6-OHDA), a sympathetic neurotoxin, was used to destroy sympathetic nerves in the islet regions. 6-OHDA produced a marked decrease (60.2%) in VMAT2-positive fibres in the islets compared to that seen with PBS treatment (Supplementary Figure S7a; n = 6 per group, *p < *0.05), indicating that 6-OHDA administration ablated sympathetic nerves in islet areas and confirming effective SpX in the pancreas. Furthermore, 6-OHDA also reduced both neuropeptide Y (NPY)- and galanin-immunoreactive nerve terminals in the islets compared to that observed with PBS treatment (Supplementary Figure S7b and S7c). In addition, SpX inhibited the GLP-1-induced amelioration of β-cell mass enlargement by HFD feeding (Fig. 4a and b; n = 6 per group, *p < *0.05). These data suggest that the effect of GLP-1 (7–36) treatment on obesity-induced alterations in β-cells in the pancreas is mediated by pancreatic efferent nerves as well as hepatic afferent nerves. Moreover, an IPGTT was performed to evaluate the effect of SpX on the alteration in glucose metabolism induced by GLP-1 (7–36). HFD feeding resulted in a significant reduction in glucose tolerance compared with that observed with Standard feeding, whereas no significant difference in fasting glucose or insulin levels was observed between any groups at baseline (Fig. 4c; n = 6 per group, *p < *0.05). Chronic GLP-1 (7–36) administration inhibited the HFD-induced increase in blood glucose at 30 min and plasma insulin concentrations at 30 and 60 min after glucose loading (Fig. 4c; n = 6 per group, *p < *0.05). However, GLP-1 (7–36) treatment had no effect on glucose tolerance in the SpX-treated groups, although SpX alone also elevated the blood glucose level at 30 min after loading. These results indicate that the improvement in glucose metabolism induced by GLP-1 treatment is mediated by pancreatic efferent nerves.

### Effects of VgX and SpX on GLP-1 receptor (GLP-1R) expression in the liver and pancreas during chronic GLP-1 (7–36) treatment

The expression of GLP-1R in the pancreas and liver was investigated using western blotting. No significant differences in the expression of GLP-1R in the liver or pancreas were observed across all groups (Supplementary Figure S8 and Supplementary Figure S9). VgX and SpX together did not affect the expression of GLP-1R, regardless of GLP-1 (7–36) treatment.

### Effects of VgX on c-fos expression in the hypothalamus, β-cell mass in the pancreas and IPGTT during chronic liraglutide treatment

**Figure 5:**
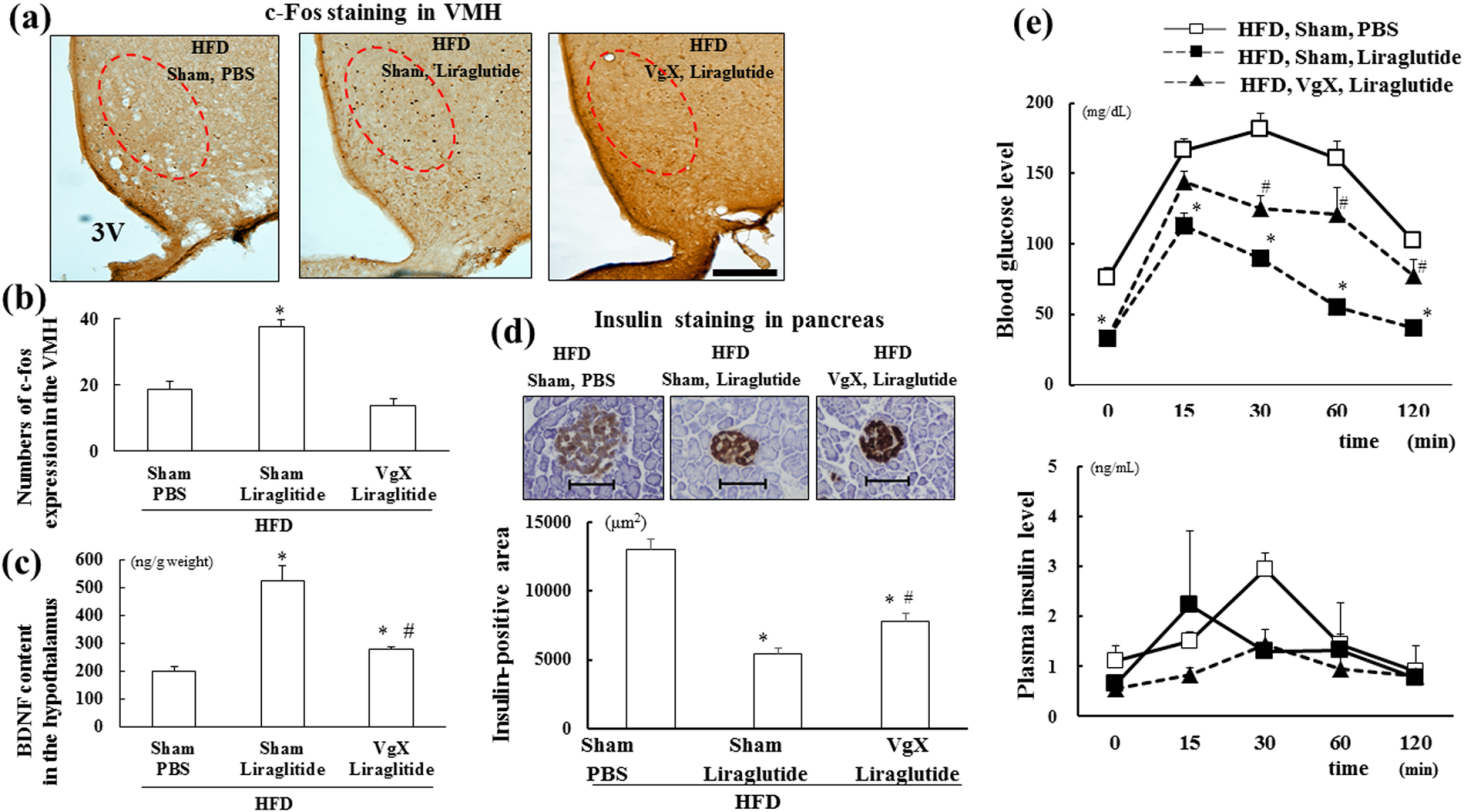
Chronic administration of liraglutide increases c-fos expression and BDNF content in the hypothalamus, restores β-cell mass and improves glucose metabolism through hepatic afferent nerves. (**a**) Representative c-fos staining performed on brain sections derived from each group. Red circle indicates the VMH area in the hypothalamus. 3 V; third ventricle. Scale bar = 100 μm. (**b** and **c**) Quantification of c-fos expression in the VMH (**b**) and BDNF content in the hypothalamus (**c**) of each group (n = 6). (**d**) Representative insulin staining performed with pancreas sections and quantification of the insulin-positive area from each group (n = 6). Scale bar = 100 μm. (**e**) Blood glucose (upper) and plasma insulin (lower) levels during the glucose tolerance tests in each group (n = 6). *p < 0.05 vs. HFD (Sham, PBS) group, ^#^ *p* < 0.05 vs. HFD (Sham, Liraglutide) group. Treatments: PBS; intraperitoneal administration of PBS, Liraglutide; intraperitoneal administration of liraglutide, Sham; sham operation, VgX; hepatic afferent vagotomy. HFD; fed with high-fat diet.

We also investigated whether liraglutide, a long-acting GLP-1R agonist, affects the hypothalamus and pancreas in a similar manner as GLP-1(7–36) treatment itself. Fasting glucose levels were lower in all of the liraglutide-treated groups than in the PBS-treated group, although there were no significant differences in daily food intake or BW among all groups (Supplementary Table 3). VgX completely inhibited the elevation of c-fos expression in the VMH (Fig. 5a and b; n = 6 per group, *p < *0.05) and partially attenuated the increase in hypothalamic BDNF content (Fig. 5c; n = 6 per group, *p < *0.05) during chronic liraglutide treatment, indicating that this GLP-1R agonist also activates the hypothalamus through hepatic afferent nerves. VgX also diminished the liraglutide-induced improvement in obesity-induced β-cell mass enlargement (Fig. 5d; n = 6 per group, *p < *0.05), suggesting that the suppressive effect of liraglutide treatment on obesity-induced β-cell proliferation is mediated by hepatic afferent nerves. An IPGTT was also performed to evaluate the effect of VgX on glucose metabolism during chronic liraglutide treatment. The fasting blood glucose levels were lower than 50 mg/dl in the liraglutide-treated groups because we performed the IPGTT after an overnight fast and a very low dose of liraglutide may remain in the body (liraglutide has an approximate half-life of 13 hr). VgX attenuated the liraglutide-induced decrease in blood glucose at 30, 60, and 120 min after glucose loading without changing the plasma insulin levels (Fig. 5e; n = 6 per group, *p < *0.05). These results indicate that the effect of liraglutide, which improves glucose metabolism in obesity, might be partially mediated by hepatic afferent nerves and the hypothalamus.

## Discussion

The major novel findings of the present study are as follows: (i) chronic administration of GLP-1 (7–36) and/or a GLP-1R agonist inhibits the obesity-induced increase in β-cell mass and proliferation; (ii) these treatments activate both the hepatic afferent nerves and pancreatic efferent nerves through the central nervous system (CNS); (iii) this neural relay system is involved in the activation of neurons in the hypothalamus and adrenergic signalling to the pancreas. Thus, this system might play an important role in preserving β-cells in obesity.

β-Cell proliferation is regulated by various metabolic demands including, peripheral insulin resistance, obesity, and hyperglycaemia^14–16^, and HFD feeding causes an increase in β-cell mass and proliferation^17, 18^. In fact, treatment with GLP-1R agonists increases β-cell proliferation in animal models of diabetes. However, the reported actions of GLP-1 are based on studies using rodent models of type 1 diabetes, including induction of diabetes through the disruption of β-cells with streptozotocin and the use of Zucker diabetic rats, whose β-cells are augmented^19^. Here, we showed that GLP-1 treatment suppressed the HFD-induced increase in β-cell proliferation. In addition, other researchers have found that several regulators of the GLP-1-induced signalling pathway inhibit β-cell proliferation and have reported that hyperplasia of pancreatic islets in HFD-fed mice is diminished by knocking out the dipeptidyl peptidase-4 (DPP-4) gene^20, 21^. Thus, GLP-1 does not necessarily stimulate β-cell proliferation.

We found that obesity-induced alterations in β-cell mass and proliferation were inhibited by chronic GLP-1 (7–36) or liraglutide treatment without altering food intake or body weight. Additionally, VgX altered these effects, indicating that chronic peripheral treatment with GLP-1 (7–36) or liraglutide normalized β-cells mass in the pancreas through the hepatic afferent nerve. VgX completely suppressed the alterations in β-cell mass induced by chronic GLP-1 (7–36) administration, whereas it only partially attenuated the increase in proliferation induced by chronic liraglutide treatment. Considering that treatment with the long-acting GLP-1R agonist, liraglutide, resulted in levels 6 to 10 times greater than endogenous GLP-1 levels, liraglutide is suggested to inhibit the obesity-induced enlargement of β-cell mass both directly through the systemic circulation and indirectly through its actions on hepatic afferent nerves.

However, there are several problems with the selectivity of VgX in our experimental manipulations. First, the afferent hepatic branch innervates not only the brain but also parts of the stomach, pyloric sphincter, pancreas and proximal duodenum, which has been completely ignored in the interpretation of the physiological experiments using VgX. Changes in specific physiological parameters, such as enzyme activities following VgX, may not necessarily result from cutting the nerves from the liver but could be the result of changes that occur with the ‘inactivity’ in the stomach or duodenum. To avoid these problems, other vagotomies will need to become more selective, or alternative approaches must be developed. Because we have been unsuccessful in our attempt to record the electrophysiological afferent activity in nerves derived from the liver, cutting the dorsal sub-diaphragmatic trunk was performed as a control procedure for VgX. We found that a few labelled cells in the DMN were observed in animals with the section of the dorsal sub-diaphragmatic trunk compared to VgX, indicating that the certain selectivity is recognized. Second, some of the afferent vagal innervations of the liver originate in the right nodose ganglion and project through the celiac branch and the periarterial plexus of the common hepatic arteries^22^. This projection cannot be easily cut in a selective manner, which cannot be completely ignored, although the contribution of afferent fibres to the liver by this route is only approximately 10%. Third, approximately 30% of the fibres in the common hepatic branch are of non-vagal origin^23^. In this context, the reliable sectioning of the proper afferent nerves that project only to the hepatoportal area in the hepatic branch has not been technically feasible in our hands.

In addition, we examined whether SpX affects GLP-1-induced alterations in β-cell mass. Administration of 6-OHDA is commonly used to selectively deplete sympathetic adrenergic neurons^24^. 6-OHDA is transported by VMAT2, which is localized at the nerve endings of sympathetic neurons, and destroys the affected nerve fibres by producing reactive oxygen species, thereby inhibiting sympathetic signal transduction^25^. We confirmed that SpX was successfully performed by showing that the 6-OHDA treatment induced a greater than 60% reduction in the islet VMAT2-positive fibre area in islets compared with that in controls, consistent with results from a previous study that validated SpX^26^. SpX through 6-OHDA treatment inhibited the GLP-1-induced improvement in β-cell enlargement induced by HFD feeding, although 6-OHDA itself did not affect β-cell mass. β-Adrenergic agonists have been shown to restore islet morphology and glucose tolerance in SpX-treated animals, and adrenalines affect islet function by stimulating β_2_-adrenoreceptors and inhibiting α_2_-adrenoreceptors on β-cells^27, 28^. Additionally, administration of 6-OHDA elevates blood glucose levels, and short exposure of non-neural cultured tissue to 6-OHDA retards the growth of cells^29, 30^. Furthermore, selective depletion of sympathetic adrenergic neurons with 6-OHDA reduces islet insulin mRNA, deteriorates islet architecture and impairs glucose tolerance in mice, which is consistent with the impaired glucose tolerance caused by 6-OHDA administration observed in this study^29, 30^. Moreover, another study observed that the peripheral administration of 6-OHDA caused a reduction in body weight for up to 3 days, just as 6-OHDA treatment significantly decreased the body weight for a week after the treatment in our study. However, this difference in body weight disappeared in the following two weeks, indicating that 6-OHDA treatment does not cause adverse effects^31^. But this SpX method raises a question regarding the comprehensive interpretation of our present results. The extent at which neurotransmitters may be released from sympathetic nerve terminals of islets is not known, but it may be assumed to occur at a low rate. The possibility that neurotransmitters other than adrenaline might contribute to the requirement of sympathetic nerve terminals for the preservation of islets cannot be excluded. For example, a population of sympathetic nerve terminals within the islets is known to harbour neurotransmitters such as NPY and galanin^32^. In fact, 6-OHDA treatment eliminated islet NPY-immunoreactive fibres, an independent marker of sympathetic fibres in pancreatic islets^33, 34^. In the present study, NPY-immunoreactive nerve terminals were reduced after chemical SpX, which is compatible with previous studies in the whole mouse pancreas showing that part of the NPY immunoreactivity is localized to sympathetic nerves^35^. Similarly, the neuropeptide galanin has been demonstrated to be partly localized to sympathetic islet nerves of the mouse pancreas, and we found that the number of galanin-immunoreactive nerve terminals was also decreased after 6-OHDA treatment^36^. Therefore, the preservation of islets might be theoretically associated with NPY and/or galanin or some other as yet unknown neurotransmitter of the sympathetic nerve separate from the adrenergic system. In any case, pancreatic efferent sympathetic nerves may regulate β-cell mass and proliferation at least partially. Moreover, our results suggest that the insulin secretion ability is not necessarily related to β-cell mass.

We focused on the change in BDNF expression in the hypothalamus and hypothesized that hypothalamic BDNF expression may be associated with these GLP-1-induced effects, as treatment with a BDNF-neutralizing antibody inhibited the GLP-1-induced alterations in the pancreas. We showed that chronic administration of GLP-1 (7–36) as well as liraglutide elevated c-fos expression and the obesity-induced reduction in BDNF content in the hypothalamus. Furthermore, these responses were attenuated by VgX, whereas VgX itself did not affect c-fos expression or hypothalamic BDNF content. In addition, the number of c-fos-positive cells was highly correlated with the hypothalamic BDNF content, indicating that GLP-1 is associated with BDNF expression in the VMH. However, GLP-1 was not shown to activate BDNF-producing cells in the VMH. Moreover, the present study indicates that these GLP-1-induced alterations were not only due to the neural connection but also the penetration of circulating GLP-1 through the blood-brain barrier (BBB) because the long-acting GLP-1R agonist, liraglutide, increased the hypothalamic BDNF content more than native GLP-1 (7–36). Systemic infusion of a GLP-1 agonist is well known to activate c-fos expression in the VMH where GLP-1Rs are localized, and circulating GLP-1 penetrates the BBB to some extent, suggesting that GLP-1 also directly binds to GLP-1R in the VMH through crossing the BBB^37–39^. In the present study, acute GLP-1 treatment induced c-fos expression in both the PVN and VMH, whereas chronic GLP-1 (7–36) or liraglutide treatment promoted c-fos expression only in the VMH, where BDNF-expressing neurons are located. In contrast, our previous study demonstrated that acutely elevated intraportal GLP-1 levels induce c-fos expression in several discrete regions of the brain, including the PVN and VMH of the hypothalamus via hepatic afferent nerves^8^. This difference may depend on the methodology, i.e., “acute” vs. “chronic” treatment. In any case, GLP-1 at least activates neurons in the VMH, and this activation is related to the preservation of β-cell mass in obesity.

GLP-1 is well known to be able to bind GLP-1R directly in β-cells and control insulin release^40–42^. However, this study showed that GLP-1 (7–36), as well as liraglutide, improved glucose metabolism without increasing insulin secretion in the IPGTT, which is thought to provide an overall benefit to β-cells. Several studies have shown that increasing the concentration of circulating GLP-1 in patients with impaired fasting glucose (IFG) promotes insulin secretion but does not affect insulin secretion in healthy controls^43, 44^. The obese rats used in this study did not exhibit a change in fasting plasma insulin levels but showed an increase in blood glucose and plasma insulin levels after glucose loading, indicating that they are in the initial stage of glucose intolerance. These findings are compatible with a previous finding that a similar enhancement in GLP-1 by a DPP-4 inhibitor during a glucose tolerance test did not induce a significant difference in insulin secretion between IFG and normal glucose tolerance (NGT) groups. One possible explanation for these results is that the improvement in glucose metabolism induced by these GLP-1 agents might be related to a reduction in glucagon secretion via hypothalamic neuronal activation; in fact, our previous study demonstrated that intraportal administration of GLP-1 (7–36) reduces glucagon secretion through a hepatic afferent nerve-hypothalamic-pancreatic efferent nerve pathway, although the reason both GLP-1 (7–36) and liraglutide treatment did not stimulate insulin release is uncertain^13^. We also found that chronic administration of liraglutide but not GLP-1 (7–36) reduced fasting glucose levels regardless of whether the neural connection was dissected or not. This difference in fasting glucose levels between liraglutide and GLP-1 (7–36) treatment is probably due to the greater pharmacological potency of liraglutide to activate GLP-1R. Indeed, VgX caused a blood glucose change; however, liraglutide was able to completely block the VgX-induced alteration in the fasting glucose level, suggesting that liraglutide directly activates the hypothalamus through by penetrating the BBB and acting on GLP-1R and regulates glucose metabolism via efferent nerves to the pancreas^45^. Moreover, GLP-1 has been reported to influence blood glucose levels by decreasing hepatic gluconeogenesis^44^. Considering that hepatic insulin resistance is frequently seen even in the pre-diabetic state and is a major cause of the high level of gluconeogenesis, the glucose-lowering effect, which is independent of insulin secretion, might occur with liraglutide treatment by decreasing glucagon secretion and hepatic glucose production, suggesting that GLP-1 may not necessarily regulate insulin release^46^.

Furthermore, we speculated that HFD feeding and/or denervation would alter GLP-1R expression in the pancreas and liver. Nevertheless, we observed that neither HFD feeding nor denervation affected GLP-1R expression in the pancreas or liver, demonstrating that alterations in glucose metabolism due to HFD feeding and/or denervation may not be directly associated with the expression of GLP-1R.

**Figure 6:**
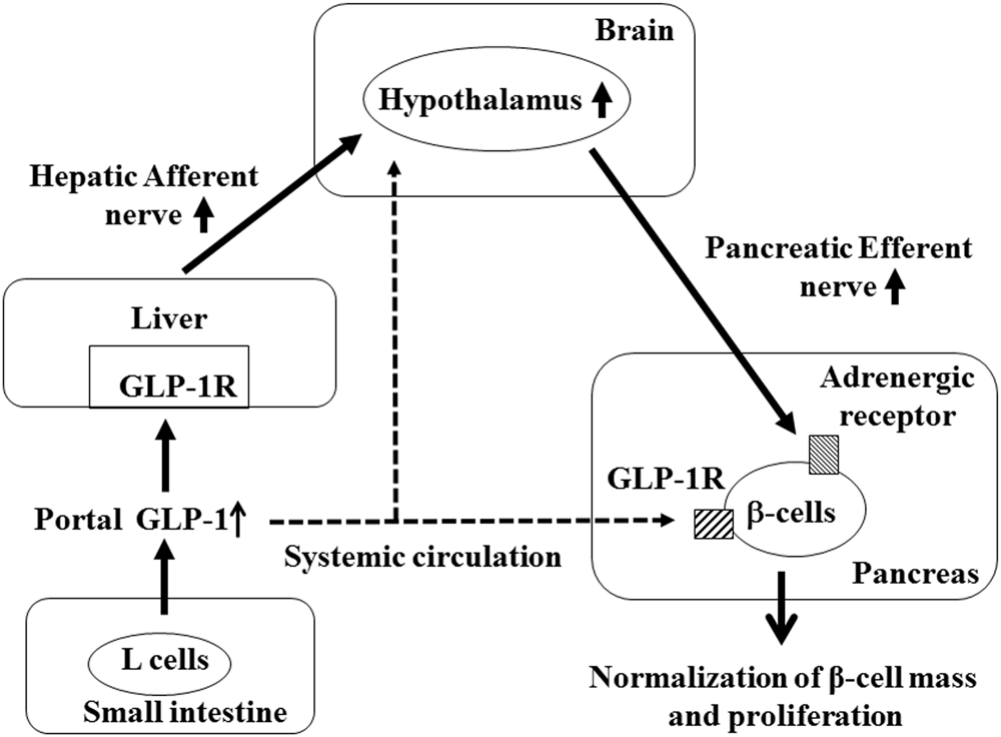
Schematic model of the neuronal relay mediating intra-organ communication. GLP-1 secreted from L cells in the small intestine stimulates the hypothalamus indirectly through the activation of hepatic afferent nerves and directly through crossing the BBB. Activated hypothalamic neurons stimulate efferent nerves innervating the pancreas, which modulate β-cells via adrenergic receptors. In addition, GLP-1 also acts directly on β-cells through GLP-1R.

In summary, the essential link between the neural pathways and an augmentation of islet preservation by peripheral administration of GLP-1 was shown in the present study. GLP-1 acts directly on β-cells in the pancreas through the systemic circulation; additionally, GLP-1 stimulates the CNS through hepatic afferent nerves and, in turn, activates pancreatic efferent nerves and preserves β-cells at least partially via adrenergic signalling (Fig. 6).

## Conclusions

GLP-1 normalizes the obesity-induced compensatory increase in β-cell mass and proliferation through a neuronal relay system that consists of afferent and efferent autonomic nerves, and the CNS and plays an important role in inter-organ (liver to pancreas) communication. Further investigation is required to determine whether these findings are attributable to the GLP-1-related network that involves the liver, brain, and pancreas.

## Materials and Methods

### Animals

Male Sprague–Dawley rats (250–280 g; Seac Yoshitomi, Fukuoka, Japan) were housed in a room with daily illumination from 07:00 to 19:00 (12-h/12-h light/dark cycle) and were maintained at 21 ± 1 °C with 55 ± 5% humidity. Animals were provided *ad libitum* access to standard chow (Clea chow, Clea, Tokyo, Japan) and tap water. All experiments were performed in accordance with the guidelines established by the National Institutes of Health, USA, regarding the care and use of animals for experimental procedures. Additionally, the ethics committee of the Division of Laboratory Animal Science, Research Promotion Project of Oita University specifically approved this study.

### Reagents

On the day of the experiment, GLP-1 (7–36) (Sigma, St. Louis, MO, USA) or liraglutide (Peptides International Inc., Louisville, KY, USA) was freshly dissolved in 0.1 M phosphate-buffered saline (PBS), adjusted to pH 7.0.

### Vagotomy (VgX)

Rats were anaesthetized with sodium pentobarbital (50 mg/kg, intraperitoneal; ip), and a midline laparotomy was performed. The common hepatic vagal branch was selectively transected by stretching the fascia containing the common hepatic vagal branch. For sham-operated animals, the hepatic vagal branch was only exposed^6^. The hepatic branch of the vagal nerve was selectively sectioned (vagotomy; VgX) in experiments conducted to determine the role of vagal innervation. Cutting only the dorsal sub-diaphragmatic trunk was performed as a control procedure for the VgX. During all procedures, the abdominal cavity was bathed with saline to prevent drying of the viscera. This operation was performed according to the same method described in our previous research that showed that VgX suppresses the intraportal GLP-1 elevation-induced anorexic effect and c-fos expression in several areas of the brain^8, 13^. To verify the selectivity of the VgX, a Fluorogold tracer strategy was used^47^. Animals treated with Sham, VgX and sectioning of the dorsal sub-diaphragmatic trunk received an intraperitoneal injection of 1 mg of Fluorogold (Fluorochrome LLC, CO, USA) in 1 ml of PBS. Five days later, each animal was given a lethal dose of pentobarbital sodium and then transcardially perfused with 100 ml of PBS at 40 °C followed by 100 ml of 4% paraformaldehyde in PBS. Fluorogold was examined with an epifluorescence microscope, and accuracy of the VgX was assessed by the absence or presence of the appropriate columns of preganglionic neurons in the dorsal motor nucleus (DMN) of the vagus (Supplementary Figure S3A).

### Sympathectomy (SpX)

To destroy the sympathetic nerves in the islet regions, a single ip administration of the sympathetic neurotoxin, 6-hydroxydopamine (6-OHDA; 50 mg/0.1 ml/kg body weight (BW) in 20% ascorbic acid, Sigma) was performed. Confirmation of the efficacy of pharmacological sympathectomy (SpX) was performed by determining islet sympathetic innervation using immunohistochemistry for the nerve terminal marker vesicular monoamine transporter 2 (VMAT2), a transporter expressed in the synaptic vesicles of sympathetic nerves.

### Experimental protocol

Design 1: A total of 42 rats fed a standard diet (Standard: 10% fat, 70% carbohydrate, 20% protein; Clea chow) were divided into six groups of six rats, each matched for daily food intake and BW. Two groups underwent VgX, and the other groups were sham-operated. After an overnight fast, the sham-treated groups were sacrificed 2 h after ip administration of GLP-1 (7–36) [1.25, 2.5, 5, or 10 nmol/kg BW] or PBS instead of GLP-1 (7–36) (n = 6 per group). The VgX-treated groups were also sacrificed 2 h after GLP-1 (7–36) treatment (0 nmol/kg BW or 5 nmol/kg BW; n = 6 per group).

Design 2: Thirty rats were assigned to one of five groups (n = 6 per group). In Group 1, rats were fed the standard diet (Standard) for 3 weeks. The sham operation was performed 1 week before chronic treatment with PBS, and then rats were fed the Standard for 3 more weeks. In Group 2, rats were fed a HFD (60% fat, 20% carbohydrate, 20% protein; Diet Research, New Brunswick, NJ, USA) for 3 weeks. The sham operation was performed 1 week before chronic treatment with PBS, and then rats were fed the HFD for 3 more weeks. In Group 3, rats were fed the HFD for 3 weeks. The sham operation was performed 1 week before chronic treatment with GLP-1 (7–36) (5 nmol/kg BW), and then rats were fed the HFD for 3 more weeks. In Group 4, rats were fed the HFD for 3 weeks. VgX was performed 1 week before chronic treatment with PBS, and then rats were fed the HFD for 3 more weeks. In Group 5, rats were fed the HFD for 3 weeks. VgX was performed 1 week before chronic treatment with GLP-1 (7–36) (5 nmol/kg BW), and then rats were fed the HFD for 3 more weeks. GLP-1 (7–36) or PBS was administered by ip injection twice daily (at 7 am and 7 pm) for 3 weeks (Supplementary Figure S1).

Design 3: Thirty rats were assigned to one of five groups (n = 6 per group). In Group 1, rats were fed the Standard for 3 weeks, were chronically given PBS for 3 more weeks, and then were fed the Standard. A single ip administration of PBS was performed 1 week before chronic treatment with PBS. In Group 2, rats were fed the HFD for 3 weeks and were then chronically given PBS for 3 more weeks. A single ip administration of PBS was performed 1 week before chronic treatment with PBS, and then rats were fed the HFD. In Group 3, rats were fed the HFD for 3 weeks, were chronically given GLP-1 (7–36) (5 nmol/kg BW) for 3 more weeks, and were then fed the HFD. A single ip administration of PBS was performed 1 week before chronic treatment with GLP-1 (7–36). In Group 4, rats were fed the HFD for 3 weeks, were chronically given PBS for 3 more weeks, and were then fed the HFD. A single ip administration of 6-OHDA was performed 1 week before chronic treatment with PBS. In Group 5, rats were fed the HFD for 3 weeks, were given GLP-1 (7–36) (5 nmol/kg BW) for 3 more weeks, and were then fed the HFD. A single ip administration of 6-OHDA was performed 1 week before chronic treatment with GLP-1 (7–36). Chronic administration of GLP-1 (7–36) or PBS was performed by ip injection twice daily (at 7 am and 7 pm) for 3 weeks. We show outlines of this study over the course of the experimental period (Supplementary Figure S1).

Design 4: On the day of the experiment, liraglutide was freshly dissolved in 0.1 M PBS. Rats were divided into three groups (n = 6 per group). In Group 1, rats were fed the HFD for 3 weeks. The sham operation was performed 1 week before chronic treatment with PBS, and then rats were fed the HFD for 3 more weeks. In Group 2, rats were fed the HFD for 3 weeks. The sham operation was performed 1 week before chronic treatment with liraglutide (10 µg/kg BW), and then rats were fed the HFD for 3 more weeks. In Group 3, rats were fed the HFD for 3 weeks. VgX was performed 1 week before chronic treatment with liraglutide, and then rats were fed the HFD for 3 more weeks. Chronic administration of liraglutide or PBS was performed by ip injection once daily at 7 pm for 3 weeks.

### Glucose tolerance test

After an overnight fast, all rats in Design 3 and 4 were intraperitoneally administered glucose (1.5 g/kg BW), and blood samples were taken at 0, 15, 30, 60, and 120 min. Blood samples were collected from the tail vein and immediately centrifuged at 4000 × *g* for 10 min at 4 °C. Blood glucose concentrations were measured using the glucose oxidase method and a glucose analyser (MS-GR101; Terumo, Tokyo, Japan), and plasma insulin concentrations were measured using a commercially available kit (Shibayagi, Gunma, Japan).

### Measurement of food intake, BW, and BDNF content in the hypothalamus

Food intake and BW of all animals were measured every morning during the experiments. After an overnight fast, all animals were exsanguinated following transcardiac perfusion with 100 ml saline containing 200 U heparin. Half of the hypothalamus was dissected with a sterile razor blade on dry ice, with the boundaries of the hypothalamic blocks at the optic chiasm in the rostral, at the mammillary bodies in the caudal, and at the hypothalamic sulcus in the lateral directions. The tissue was homogenized and extracted with an acid-ethanol solution (concentration ratio of HCl: ethanol: H2O = 1.5:75:23.5). The homogenates were immediately frozen and stored at −80 °C until analysis. BDNF (mature form) levels were measured in homogenates using a BDNF ELISA kit (Insight Genomics, Falls Church, VA, USA). A 50-μl aliquot of the homogenate was removed for the protein assay (Bio-Rad, Hercules, CA, USA).

### Immunohistochemistry

The pancreas was removed, post-fixed for 24 h with 4% paraformaldehyde in 0.1 M PBS, and then fixed in paraffin blocks. The tissue was sliced into 5-µm sections, deparaffinized, and subjected to heat-induced epitope retrieval, which was performed by heating of the sample in a microwave at 600 W for 1 min in citrate buffer (pH 6.0), followed by cooling at room temperature for 30 min. Sections were rinsed, and at least two slides per adult pancreas were immunohistochemically stained for a marker of cellular proliferation (Ki-67) and insulin. After endogenous peroxidase was quenched (0.5% H_2_O_2_, 0.1 M PBS, 30 min, room temperature) and non-specific interactions were blocked (4% bovine albumin, diluted in 0.1 M PBS and 0.3% Triton X-100 at room temperature), sections were incubated with polyclonal rabbit antiserum recognizing insulin (1:100, rat specific, Cell Signaling Technology, Inc., Danvers, MA, USA), Ki-67 (1:1000, rat specific, Abcam, Inc., Cambridge, MA, USA), VMAT2 (1:1000, rat specific, Abcam, Inc.), NPY (1:1000, rat specific, Abcam, Inc.), and galanin (1:500, rat specific, Abcam, Inc.) diluted in 0.1 M PBS and 0.3% Triton X-100 at room temperature. Sections were transferred to a solution containing a biotinylated secondary antibody, incubated for 30 min, rinsed, transferred to avidin-biotinylated peroxidase for 30 min, and finally developed using diaminobenzidine as a substrate (Nacalai Tesque, Kyoto, Japan) for Ki-67, NPY and galanin staining and 3-amino-9-ethylcarbazole for insulin staining. Avidin-biotinylated Alexa Fluor 488 (Life Technologies Inc., Rockville, MD, USA) was used for VMAT2 visualization.

Immunostaining for c-fos, the protein encoded by *c-fos*, to examine neuronal activation was performed with half of the brain from each group in Designs 1, 2, and 4. Half of the brain was removed and fixed in 4% paraformaldehyde in 0.1 M PBS. c-Fos expression was examined in the areas of interest [lateral hypothalamus (LH), paraventricular nucleus (PVN), ventromedial hypothalamus (VMH), arcuate nucleus (ARC)] in the hypothalamus, defined according to the atlas of Paxinos and Watson (Supplementary Figure S2)^48^. Brains sections (40 µm thick) were collected using a freezing sliding microtome and were stored in 0.1 M PBS with 0.1% sodium azide (Wako, Japan) at 4 °C. After endogenous peroxidase was quenched (0.5% H_2_O_2_, 0.1 M PBS, 30 min, room temperature) and non-specific interactions were blocked (5% bovine albumin at room temperature), brain sections were incubated with polyclonal rabbit antiserum recognizing c-fos (1:100, rat specific, Abcam, Inc.). Sections were transferred to a solution containing biotin-conjugated goat anti-rabbit antibody. Immunoreactivity for c-fos was visualized with diaminobenzidine (Nacalai Tesque). The number of c-fos-positive cells in the unilateral area of five coronal sections from each animal was calculated by observers who were blinded to the conditions of the animals and specimens. The statistical analysis was performed with the average data of the unilateral area from each animal. Normal rabbit serum was used as a negative control for Ki-67, insulin, VMAT2, and c-fos, and further incubation with a secondary antibody was performed as described above.

All islets in each section were observed using a BIOREVO BZ-9000 microscope (Keyence, Birmingham, AL, USA). The area of insulin-stained β-cells, the number of Ki-67-positive nuclei, and the VMAT2-positive fibre area were evaluated in 100 islets in three different regions of the pancreatic specimens in each animal and were calculated by using BZ-H1M software (Keyence).

### Western blotting

Hepatic and pancreatic preparations were homogenized in sample buffer and separated by centrifugation, and the supernatant was boiled for 4 min. The total protein content of the tissue was measured using the Bradford method. Equal amounts of total protein were resolved by electrophoresis on 8% sodium dodecyl sulfate–polyacrylamide gels and then electrophoretically transferred onto polyvinylidene difluoride membranes (Bio-Rad Laboratories, Hercules, CA, USA). Non-specific binding to the membranes was blocked with 5% non-fat milk for 1 h, and the membranes were subsequently incubated overnight with primary antibodies at 4 °C followed by the secondary antibody for 1 h at 20 °C. The primary antibody was the polyclonal rabbit anti-GLP-1R antibody (rat specific, Abcam, Inc.). GLP-1R was detected using enhanced chemiluminescence (Amersham Life Sciences, Arlington Heights, IL, USA). Each protein was measured using Quantity One imaging software (Bio-Rad).

### Statistics

Results are expressed as the mean ± standard error of the mean. Statistical significance was evaluated using two-way analysis of variance followed by Scheffe’s test for *post hoc* comparisons. For all tests, the level of significance was set at *p < *0.05.

## Electronic supplementary material

Supplementary information
