# Supplementary material for: Glucagon-like peptide-1 reduces pancreatic β-cell mass through hypothalamic neural pathways in high-fat diet-induced obese rats
Source: Sci Rep. 2017 Jul 17;7:5578. doi: 10.1038/s41598-017-05371-4 (PMC5514038; doi:10.1038/s41598-017-05371-4)
Supplement: Supplementary file 1 — Supplementary information [file 41598_2017_5371_MOESM1_ESM.pdf]

Supplementary information

Glucagon-like peptide-1 reduces pancreatic β-cell mass through hypothalamic neural pathways in high fat diet-induced obese rats

Hisae Ando, Koro Gotoh, Kansuke Fujiwara, Manabu Anai, Seiichi Chiba, Takayuki Masaki, Tetsuya Kakuma and [1]Hirotaka Shibata

Department of Endocrinology, Metabolism, Rheumatology and Nephrology, Faculty of Medicine, Oita University, Yufu city, Oita 879-5593, Japan

Address correspondence to:
Koro Gotoh
Department of Endocrinology, Metabolism, Rheumatology and Nephrology, Oita University, 1-1 Idaigaoka, Hasama, Yufu city, Oita 879-5593, Japan
Tel: +81-97-586-5793
Fax: +81-97-549-4480
E-mail: gotokoro@oita-u.ac.jp

	Standard	HFD			
	Sham, PBS	Sham, PBS	Sham, GLP-1	VgX, PBS	VgX, GLP-1
Daily food intake (kcal/day)	97.3 ± 4.9	98.5 ± 1.7	97.8± 1.6	99.8 ± 1.1	94.0 ± 0.9
Fasting blood glucose (mg/dl)	99.3 ± 3.5	97.8 ± 6.6	102.6 ± 2.7	89.6 ± 7.0	102.3 ± 7.0

Supplementary TABLE 1. Effect of hepatic afferent vagotomy on daily food intake and fasting blood glucose.

Treatments: Standard; fed with standard diet, HFD; fed with high fat diet, Sham; sham operation, VgX; hepatic afferent vagotomy, PBS; intraperitoneal administration of PBS; GLP-1; intraperitoneal administration of GLP-1 (7-36).

	Standard	HFD			
	Sham, PBS	Sham, PBS	Sham, GLP-1	VgX, PBS	VgX, GLP-1
Daily food intake (kcal/day)	97.9 ± 12.0	112.2 ± 12.1	101.9± 2.8	88.5 ± 5.2	88.0 ± 5.9
Fasting blood glucose (mg/dl)	73.2 ± 7.6	75.7 ± 6.3	70.0 ± 5.3	73.3 ± 7.0	69.4 ± 6.8

Supplementary TABLE 2. Effect of pancreatic efferent sympathectomy on daily food intake and fasting blood glucose.

Treatments: PBS; intraperitoneal administration of PBS; GLP-1; intraperitoneal administration of GLP-1 (7-36), Standard; fed with standard diet, HFD; fed with high fat diet, Sham; preservation of sympathetic nerve with PBS administration, SpX; pancreatic efferent sympathectomy with 6-OHDA administration.

	HFD		
	Sham, PBS	Sham, Liraglutide	VgX, Liraglutide
Body weight (g)	557.0 ± 17.8	507.8 ± 14.0	503.2 ± 26.2
Daily food intake (kcal/day)	105.7 ± 4.3	103.0 ± 2.1	97.6 ± 4.3
Fasting blood glucose (mg/dl)	100.7 ± 5.1	58.2 ± 6.5*	52.6 ± 6.5*

Supplementary TABLE 3. Effect of vagotomy and administration of liralutide on body weight, daily food intake, and fasting blood glucose.

*$p < 0.05$ vs. HFD (Sham, PBS) group. Treatments: Standard; fed with standard diet, HFD; fed with high fat diet, Sham; sham operation, VgX; hepatic afferent vagotomy, PBS; intraperitoneal administration of PBS; Liraglutide; intraperitoneal administration of liraglutide.

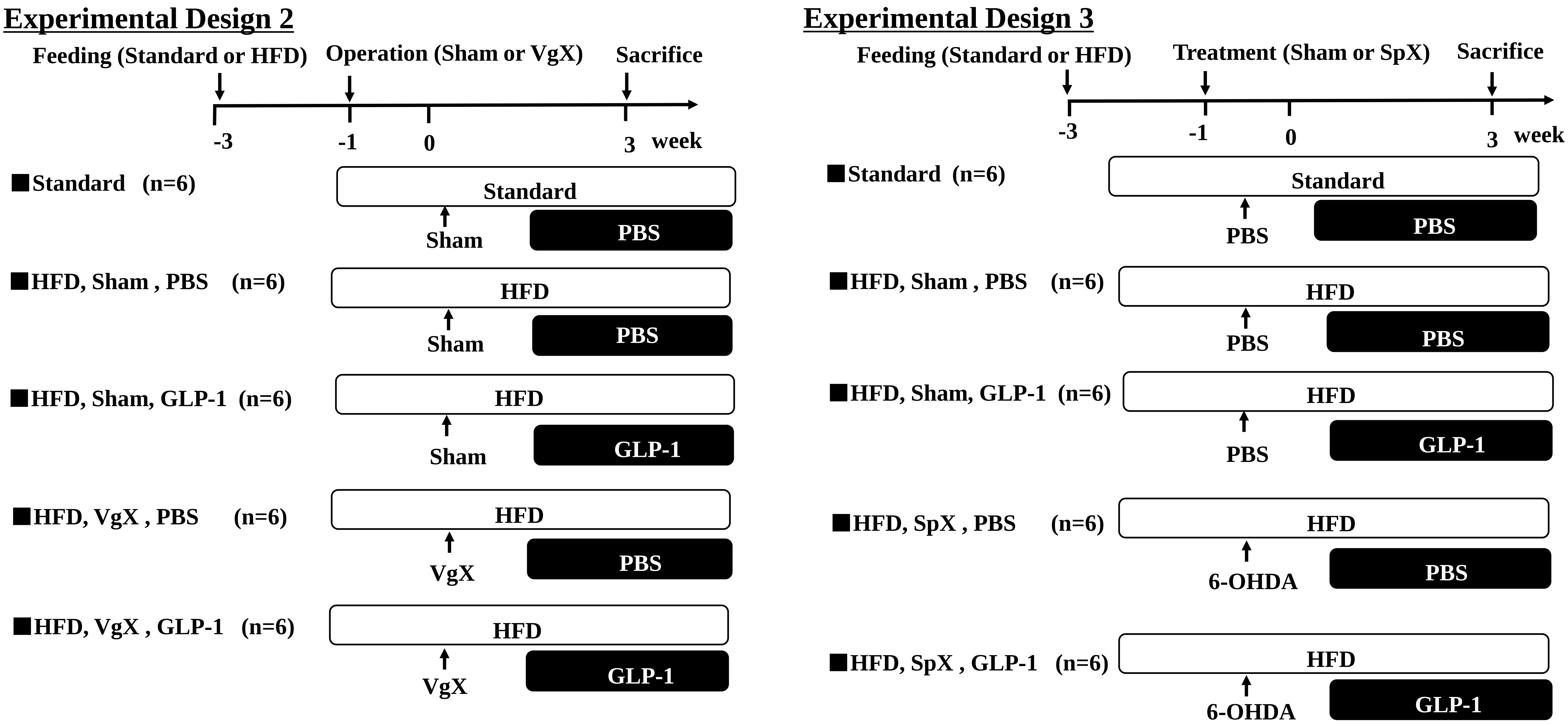

Supplementary Figure S1. Protocol of experimental Design 2 and Design 3.
Treatments: Standard; fed with standard diet, HFD; fed with high-fat diet, Sham; sham operation, VgX; hepatic afferent vagotomy, SpX; pancreatic efferent sympathectomy with 6-OHDA administration, PBS; intraperitoneal administration of PBS, GLP-1; intraperitoneal administration of GLP-1 (7-36).

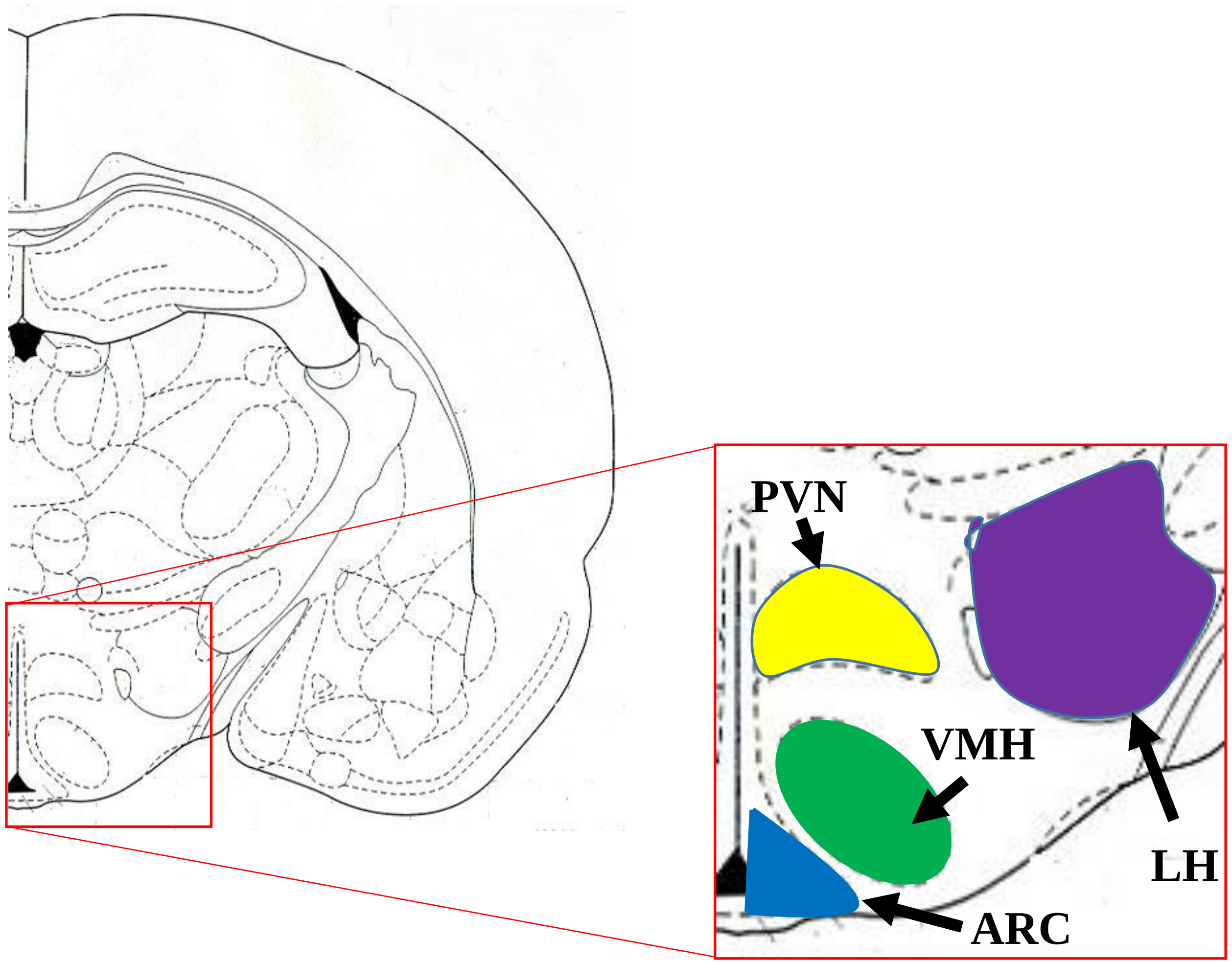

Supplementary Figure S2. Schema indicating the areas of the VMH (green), PVN (yellow), ARC (blue) and LH (purple) in the hypothalamus.

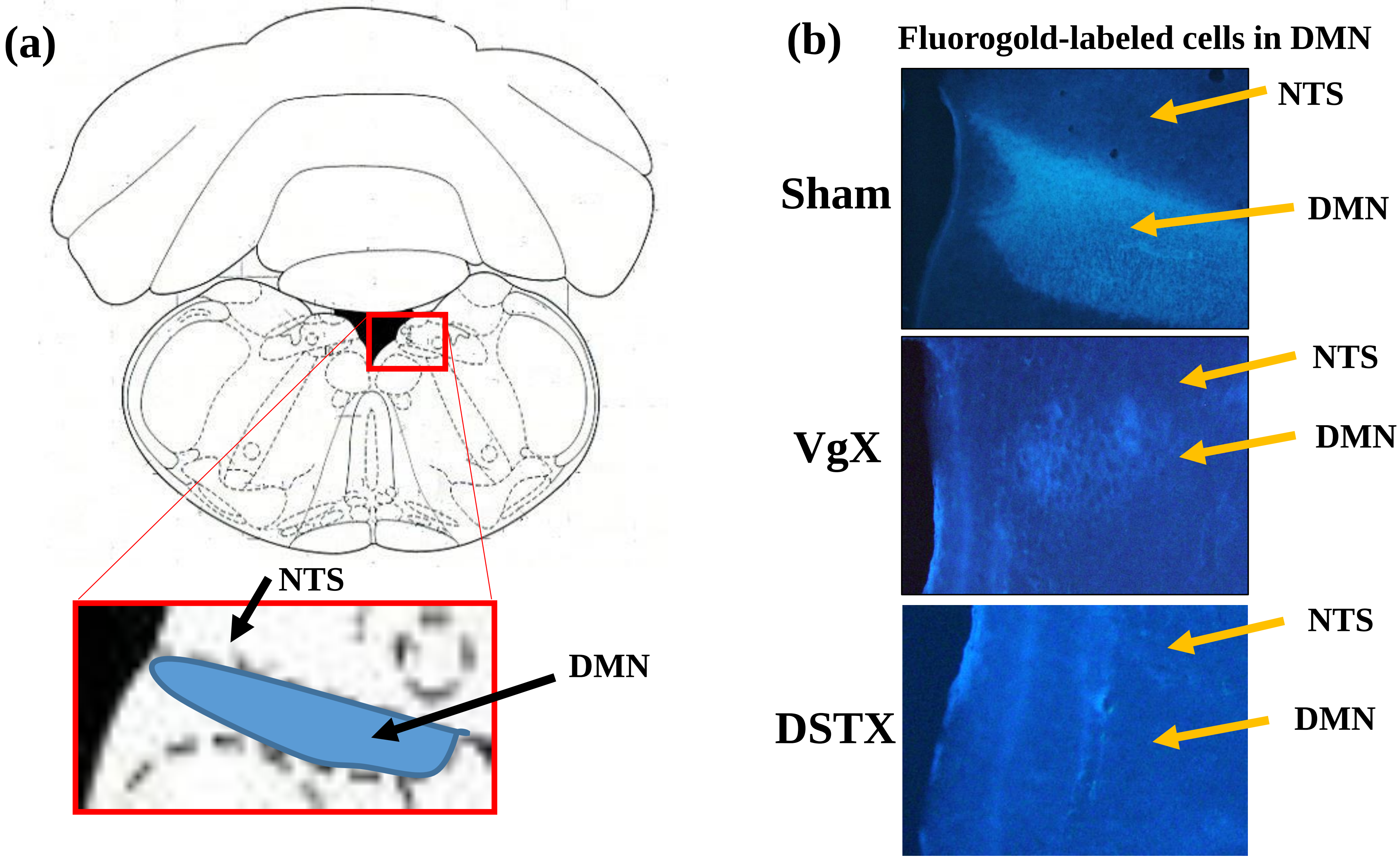

Supplementary Figure S3. Epifluorescence photomicrographs illustrating the distribution of Fluorogold-labelled cells. (**a**) Schema showing a coronal section of the DMN in the brain. (**b**) Representative labelling profiles in sham-operated (upper) and VgX-treated (lower) animals. Sham; sham operation, VgX; hepatic afferent vagotomy, DSTX; sectioning of the dorsal sub-diaphragmatic trunk, NTS; nucleus of the solitary tract, DMN; dorsal motor nucleus.

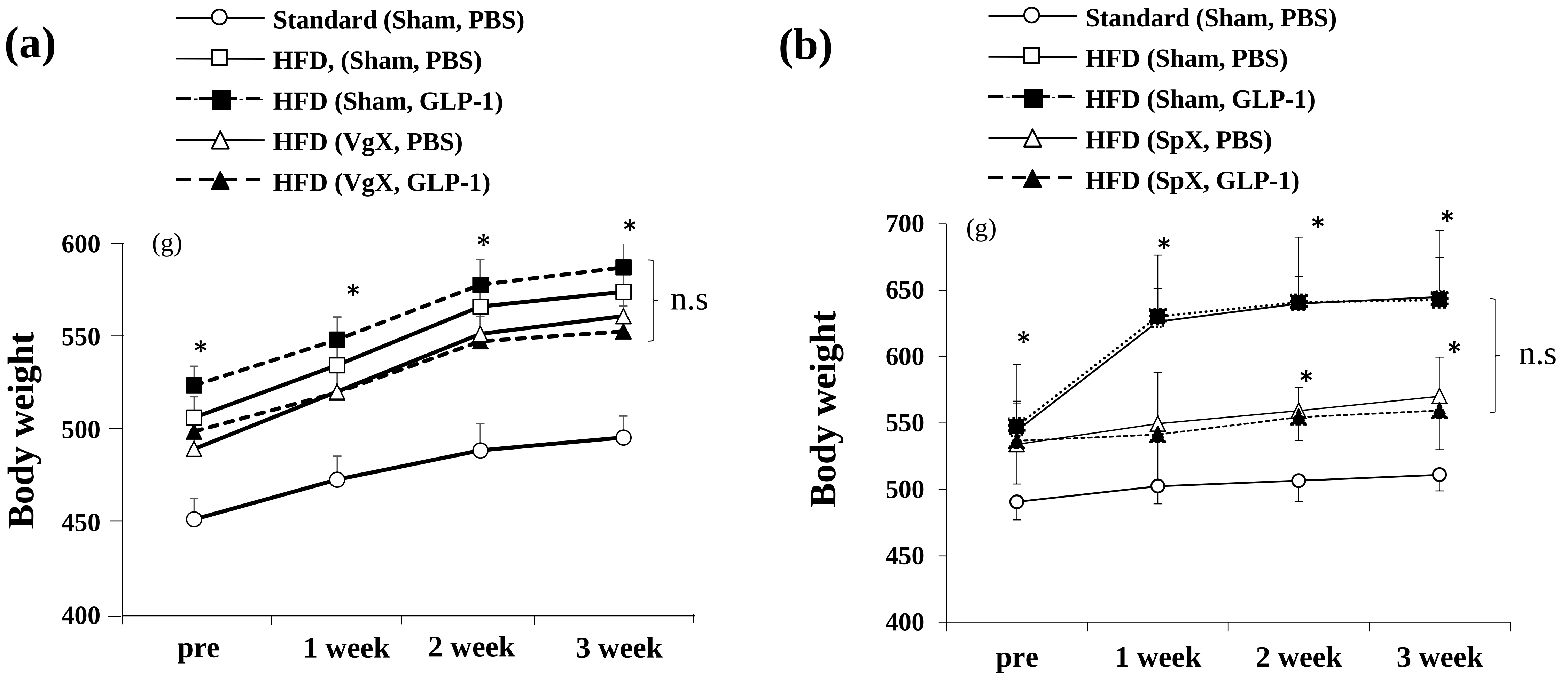

Supplementary Figure S4. Change in body weight during Experiment Design 2 and 3.
(**a** and **b**) Change in body weight during Experiment Design 2 (**a**) and Design 3 (**b**). Treatments: Standard; fed with standard diet, HFD; fed with high-fat diet, Sham; sham operation, VgX; hepatic afferent vagotomy, SpX; pancreatic efferent sympathectomy with 6-OHDA administration, PBS; intraperitoneal administration of PBS, GLP-1; intraperitoneal administration of GLP-1 (7-36). * $p < 0.05$ vs. Standard (Sham, PBS), n.s.; not significant. Pre; pretreatment with VgX or SpX.

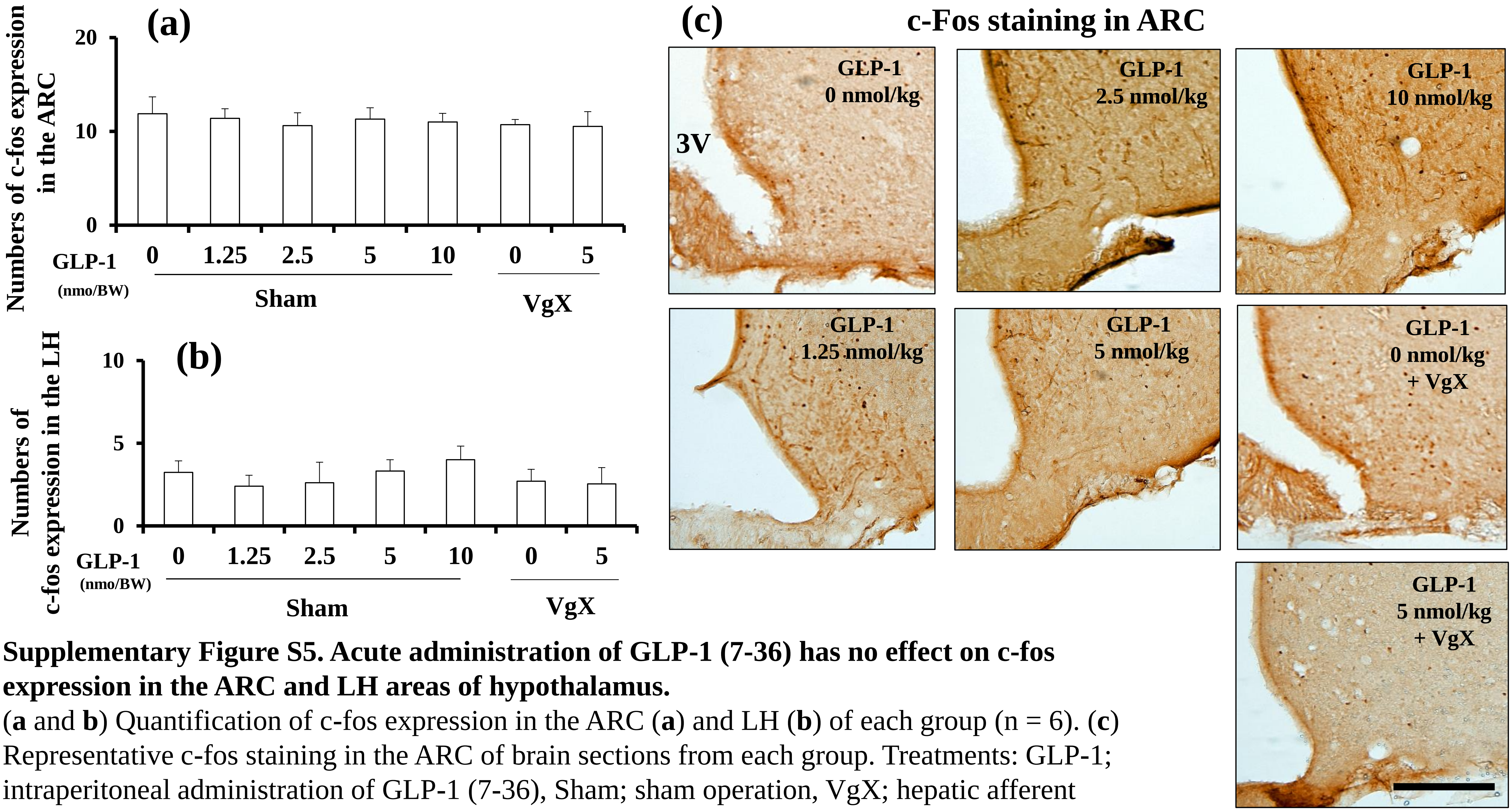

Supplementary Figure S5. Acute administration of GLP-1 (7-36) has no effect on c-fos expression in the ARC and LH areas of hypothalamus.
(**a** and **b**) Quantification of c-fos expression in the ARC (**a**) and LH (**b**) of each group (n = 6). (**c**) Representative c-fos staining in the ARC of brain sections from each group. Treatments: GLP-1; intraperitoneal administration of GLP-1 (7-36), Sham; sham operation, VgX; hepatic afferent vagotomy. Scale bar = 100 μm.

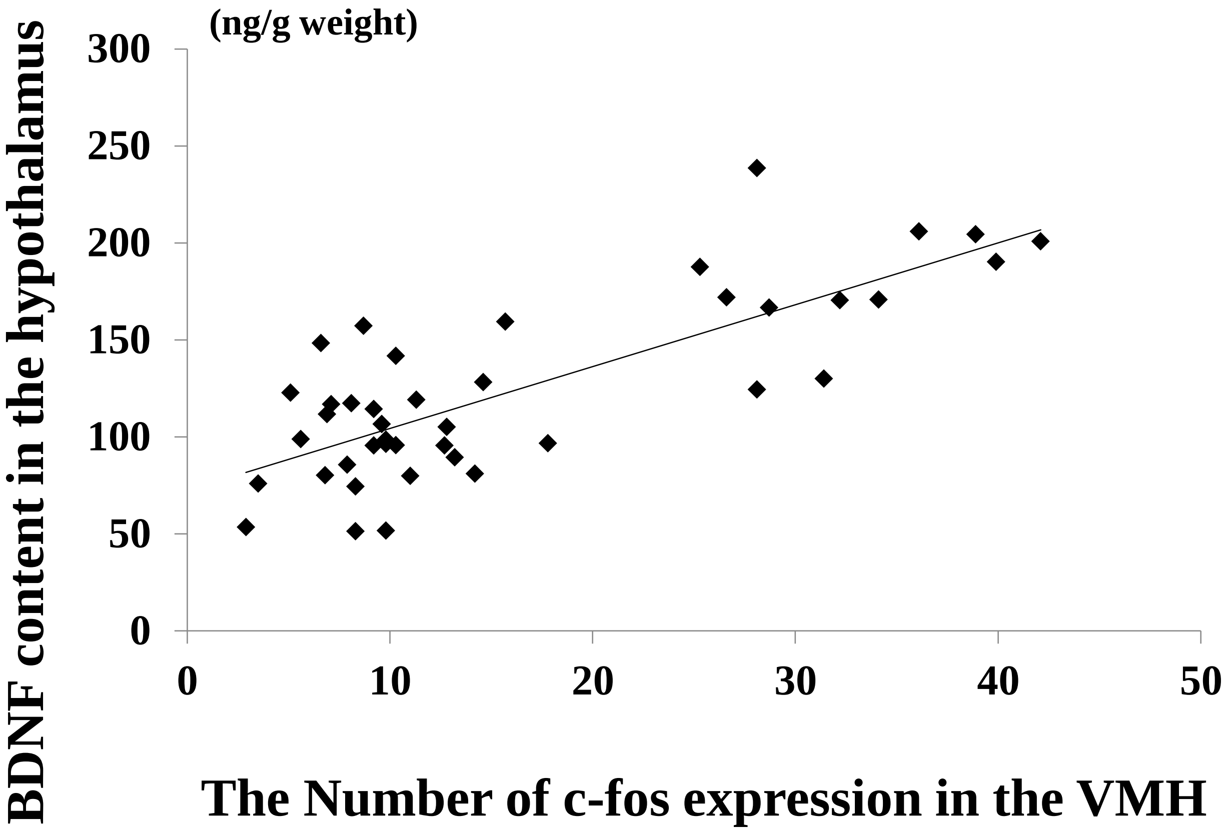

Supplementary Figure S6. Correlation between the number of c-fos-positive cells in the VMH and BDNF contents in the hypothalamus.

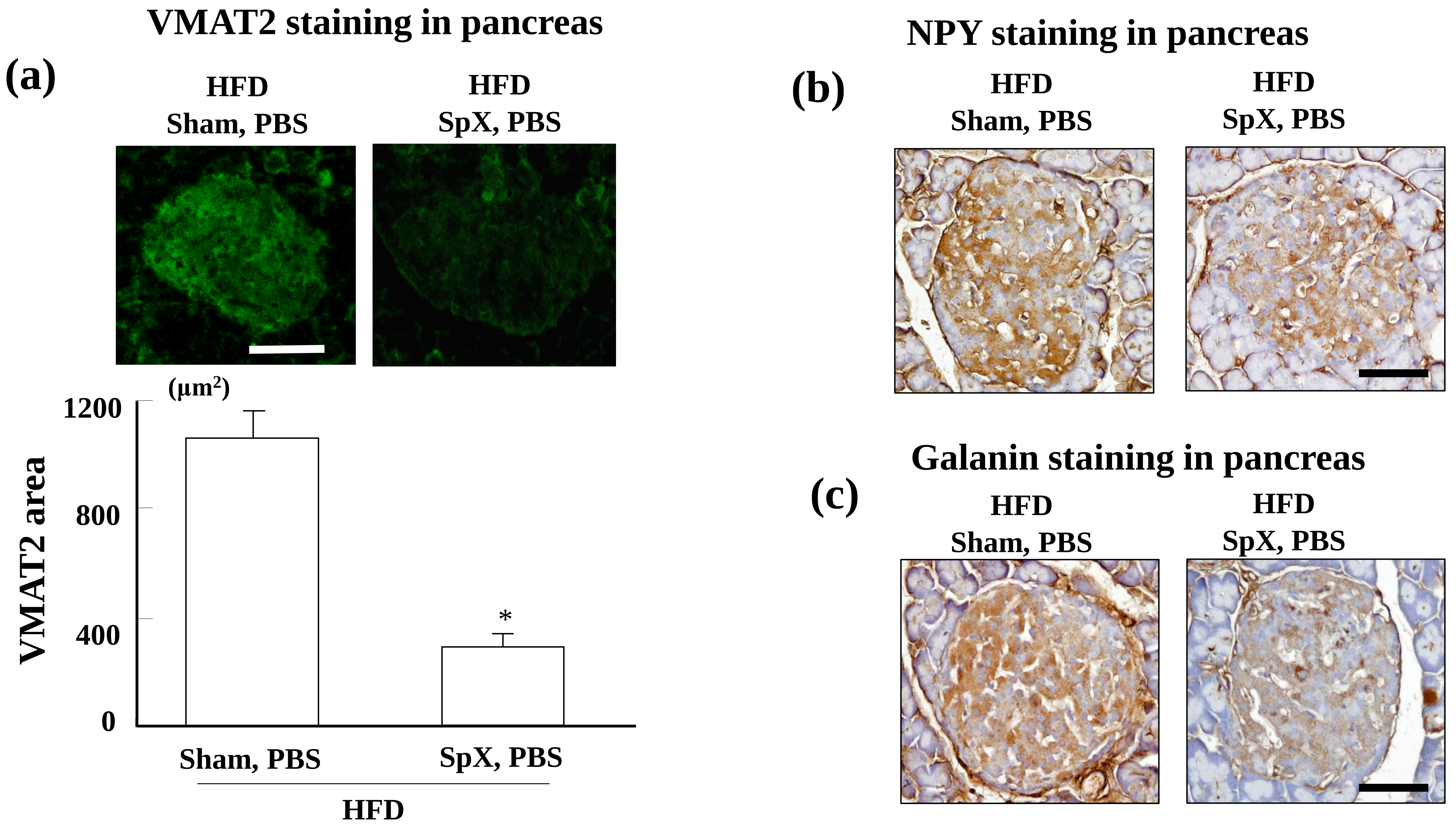

Supplementary Figure S7. Effect of 6-OHDA on islet VMAT2-positive nerve fibres and NPY- and galanin-immunoreactive nerve terminals.

Representative VMAT2 staining and VMAT2-positive areas (**a**) and representative NPY (**b**) and galanin (**c**) staining in islets of each group (n = 6). *$p < 0.05$ vs. HFD (Sham, PBS) group. Scale bar = 100 µm.

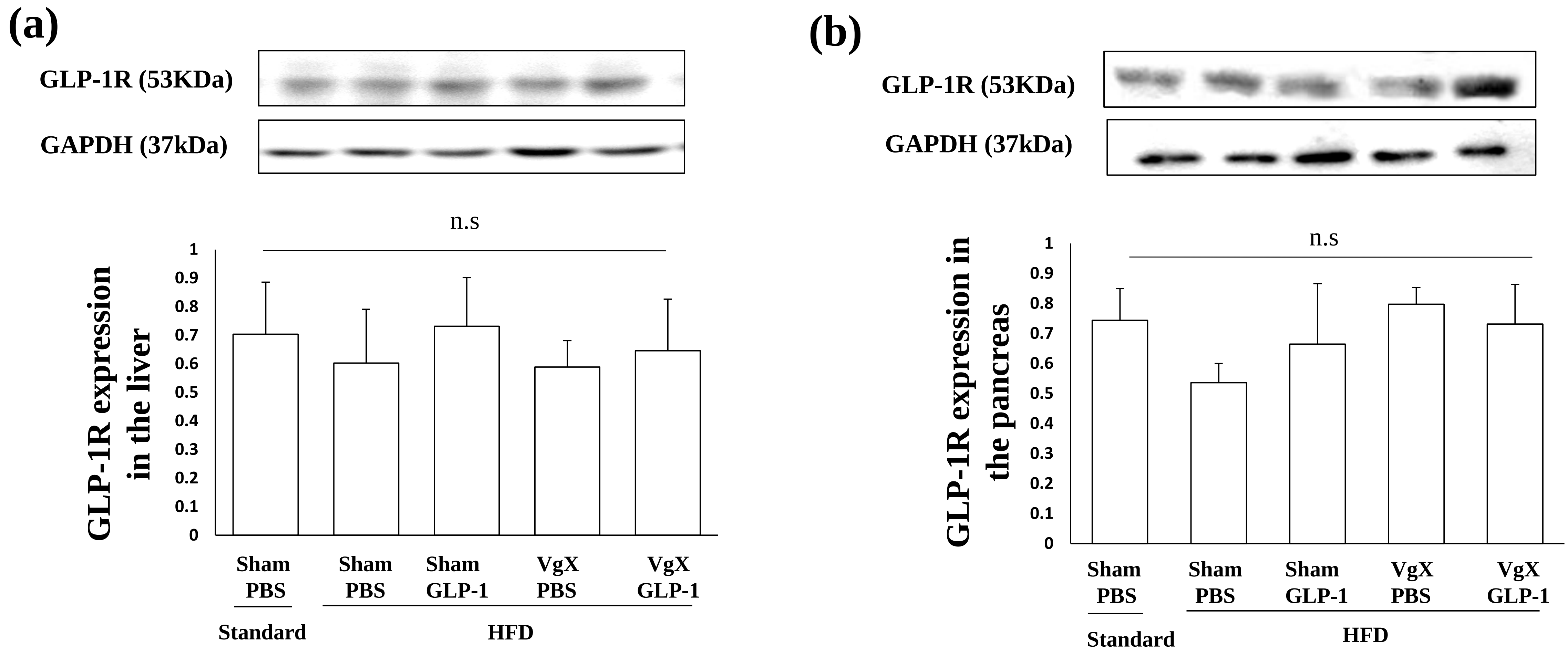

Supplementary Figure S8. VgX does not affect GLP-1R in the liver or pancreas.
(**a** and **b**) GLP-1 receptor expression in the liver (**a**) and pancreas (**b**) following VgX or Sham treatment. n.s.; not significant. Treatments: PBS; intraperitoneal administration of PBS, GLP-1; intraperitoneal administration of GLP-1, Standard; fed with standard diet, HFD; fed with high-fat diet, Sham; sham operation, VgX; hepatic afferent vagotomy. Full-length blots/gels are presented in Supplementary Figure S8.

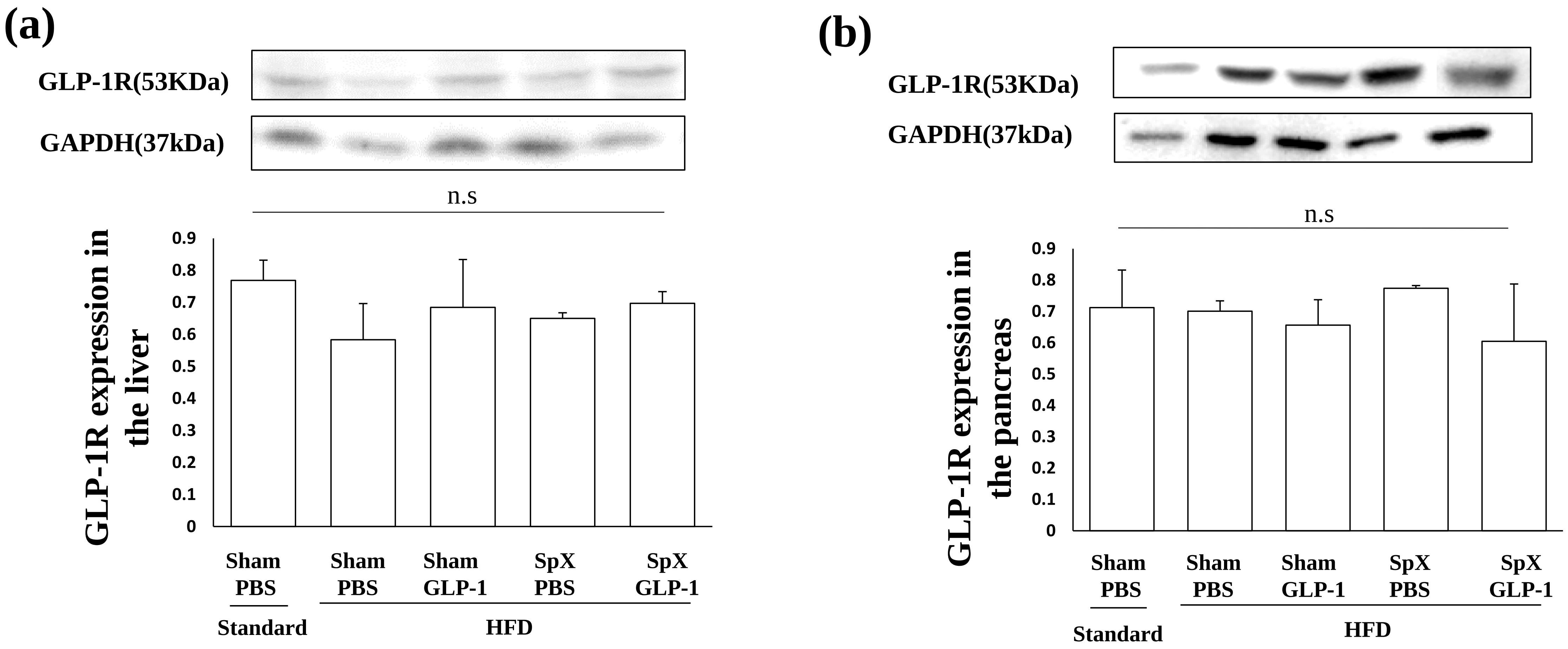

Supplementary Figure S9. SpX does not affect GLP-1R in the liver or pancreas.
(**a** and **b**) GLP-1 receptor expression in the liver (**a**) and pancreas (**b**) following SpX or Sham treatment. n.s.; not significant. Treatments: PBS; intraperitoneal administration of PBS, GLP-1; intraperitoneal administration of GLP-1, Standard; fed with standard diet, HFD; fed with high-fat diet, Sham; preservation of sympathetic nerve with PBS administration, SpX; pancreatic efferent sympathectomy with 6-OHDA administration. Full-length blots/gels are presented in Supplementary Figure S9.
